# Elucidating the structural–functional connectome of language in glioma‐induced aphasia using nTMS and DTI

**DOI:** 10.1002/hbm.25757

**Published:** 2021-12-23

**Authors:** Haosu Zhang, Sebastian Ille, Lisa Sogerer, Maximilian Schwendner, Axel Schröder, Bernhard Meyer, Benedikt Wiestler, Sandro M. Krieg

**Affiliations:** ^1^ Department of Neurosurgery Klinikum rechts der Isar, Technical University of Munich Munich Germany; ^2^ School of Medicine Technical University of Munich Munich Germany; ^3^ TUM‐Neuroimaging Center Technical University of Munich Munich Germany; ^4^ Department of Diagnostic and Interventional Neuroradiology Klinikum rechts der Isar, Technical University of Munich Munich Germany; ^5^ Center for Translational Cancer Research of the TUM (TranslaTUM) Technical University of Munich Munich Germany

**Keywords:** connectome, DTI, Glioma‐induced aphasia, graphic analysis, nTMS

## Abstract

Glioma‐induced aphasia (GIA) is frequently observed in patients with newly diagnosed gliomas. Previous studies showed an impact of gliomas not only on local brain regions but also on the functionality and structure of brain networks. The current study used navigated transcranial magnetic stimulation (nTMS) to localize language‐related regions and to explore language function at the network level in combination with connectome analysis. Thirty glioma patients without aphasia (NA) and 30 patients with GIA were prospectively enrolled. Tumors were located in the vicinity of arcuate fasciculus‐related cortical and subcortical regions. The visualized ratio (VR) of each tract was calculated based on their respective fractional anisotropy (FA) and maximal FA. Using a thresholding method of each tract at 25% VR and 50% VR, DTI‐based tractography was performed to construct structural brain networks for graph‐based connectome analysis, containing functional data acquired by nTMS. The average degree of left hemispheric networks (*M*_left_) was higher in the NA group than in the GIA group for both VR thresholds. Differences of global and local efficiency between 25% and 50% VR thresholds were significantly lower in the NA group than in the GIA group. Aphasia levels correlated with connectome properties in *M*_left_ and networks based on positive nTMS mapping regions (*M*_pos_). A more substantial relation to language performance was found in *M*_pos_ and *M*_left_ compared to the network of negative mapping regions (*M*_neg_). Gliomas causing deterioration of language are related to various cerebral networks. In NA patients, mainly *M*_neg_ was impacted, while *M*_pos_ was impacted in GIA patients.

## Graphical abstract

This is the first study to combine nTMS language mapping and connectome analysis to investigate the brain's glioma‐related functional changes; nTMS language mapping enables to investigate regions related to language superordinate controlling levels. Without sufficient compensation in glioma‐induced aphasia patients, this leads to language dysfunction. nTMS mapping can identify unstable connectomes related to functional deficits such as aphasia.

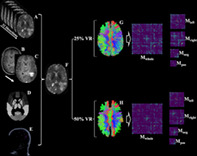

**Abbreviations**
AAT: Aachener aphasia test
AD: average degree
DCS: direct cortical stimulation
DTI: diffusion tensor imaging
EHI: Edinburgh Handedness Inventory
EL: local efficiency
EG: global efficiency
FA: fractional anisotropy
FAT: fractional anisotropy threshold
FLT: fiber length threshold
FT: fiber tracking
GIA: glioma‐induced aphasia
NEG: negative nTMS stimulation regions
nTMS: navigated transcranial magnetic stimulation
NA: no aphasia
ONT: object naming task
POS: positive nTMS stimulation regions
VR: visualized ratio

## 1 INTRODUCTION

Gliomas are the most common primary intracranial tumor, representing 81% of malignant primary brain tumors (Ostrom et al., 2013, 2014). Brain tumors within language eloquent regions can cause aphasia depending on tumor size, edema, and localization per se. However, the localization of language eloquent fiber tracts shows significant interindividual variability, especially in patients suffering from lesions in eloquent brain areas. Understanding the mechanisms of aphasia in glioma patients will improve the understanding of the neural function and structural plasticity, which is essential for preserving brain functions in individualized tumor treatment concepts.

Preoperative identification of language‐related regions is essential to preserve functionality during tumor resection in eloquent locations. Navigated transcranial magnetic stimulation (nTMS) and functional magnetic resonance imaging (fMRI) have both been widely applied preoperatively to identify the individual language‐related regions. However, fMRI lacks precision, especially in the vicinity of brain lesions based on inaccuracies due to pathological tumor vascularization (Lin et al., 2017; Silva, See, Essayed, Golby, & Tie, 2018). nTMS has shown to be highly predictive regarding negative stimulation sites, and comparisons to the gold standard of intraoperative direct cortical stimulation (DCS) showed a high accuracy of nTMS (Ille et al., 2015a, 2015b; Picht et al., 2013). Therefore, nTMS mapping of motor‐ and language‐eloquent brain areas is routinely used preoperatively to identify language‐related fiber tracts and for preoperative risk assessment combined with fiber tracking (FT).

FT is based on the fusion of data on functional brain areas such as cortical nTMS mapping data and subcortical structural tractography shown by diffusion tensor imaging (DTI; Ille et al., 2015a, 2015b). Previous studies have shown that language function is separated into cortical and subcortical collaborating networks (Hagoort, 2019; Henderson, Choi, Lowder, & Ferreira, 2016; Lohmann et al., 2010; Xiang, Fonteijn, Norris, & Hagoort, 2010). For instance, the temporo‐frontal networks are assigned to tasks on semantic and syntactic processing (Friederici, Ruschemeyer, Hahne, & Fiebach, 2003).

Connectomes are used to represent the functional composition of nodes—relevant brain areas as mapped by nTMS—and their connections as visualized by DTI within the network. The combination of graph theory, nTMS, and connectome analysis of DTI data is a novel multidisciplinary paradigm considering the brain as a complex network of individual components interacting through continuous communication. Thereby it offers further insight into both local and global effects of gliomas on these complex networks (Hart, Romero‐Garcia, Price, & Suckling, 2019). Previous studies using nTMS‐based FT only focused on single neural tracts in patients with glioma‐induced aphasia (GIA; Sollmann et al., 2020). However, recent studies have shown that gliomas have a global impact on the whole brain (Derks et al., 2017; Hart et al., 2019). Therefore, there is a necessity of analyzing global nTMS mapping‐based networks to investigate neuroplasticity and remodulation mechanisms related to aphasia.

As shown by graph theory in previous studies, the organization of cerebral structures is compatible with the hypothesis that the brain evolved to maintain the dynamic balance between maximization of the efficiency in transferring information and minimization of connection cost (Betzel et al., 2014; Bullmore & Sporns, 2009; Gargouri et al., 2016). Graph‐based network analysis enables to derive properties of the brain's “connectome” and delivers information on the topological architecture of human brain networks, such as average degree (AD), global efficiency (EG), and local efficiency (EL), which has already been introduced for functional MRI (Betzel et al., 2014) and EEG analysis (Gu et al., 2020). AD is the basic character to present the intensity of connections across all nodes in the network (Cohen & D'Esposito, 2016). EG measures the capacity in parallelly transferring and comprehensively processing information (Wang, Zuo, & He, 2010). EL indicates the fault‐tolerant capacity of the network and the efficiency of the communication between immediate neighbors of the local node (Wang et al., 2010). Previous studies on tractography networks in glioblastoma patients demonstrated significant differences in tract volumes based on nTMS positive and negative mapping sites (Sollmann et al., 2020). However, graph theory metrics and their differences have not yet been measured to analyze the impact of tumors on cerebral structural network properties. Further investigations are still lacking to distinguish the network based on eloquent language regions identified by nTMS from the left and right hemispherical networks.

This study aimed to evaluate the global and local properties of function‐specific connectomes derived from nTMS language mapping. Graph properties of aphasic and nonaphasic glioma patients of the resulting connectomes based on positive and negative nTMS mapping regions were analyzed and correlated with different states of aphasia. Connectomes were constructed and analyzed based on tractography thresholding at different levels to assess the robustness and efficacy of networks.

## 2 MATERIALS AND METHODS

### 2.1 Ethics

The current study was performed in accordance with the Declaration of Helsinki and its later amendments, and its protocol was approved and supervised by the local ethics board (registration number: 222/14, 338/16, 2793/10, 5811/13, 223/14, and 336/17). Written informed consent was obtained from all patients before enrolling in the current study.

### 2.2 Study eligibility

The following inclusion criteria were considered: (a) age above 18 years, (b) mother tongue German, (c) primary diagnosis being glioma with following pathological confirmation, (d) tumor within a left perisylvian region adjacent to the arcuate fasciculus‐related cortical and subcortical regions, (e) no previous cranial surgery, and (f) written informed consent. Patients with contraindications for MRI or nTMS examinations such as pregnancy, intracranial metallic implants, cochlear implants, and pacemakers were excluded. Overall, 30 patients with no aphasia (NA) and 30 patients with GIA were considered eligible from our database of patients undergoing nTMS language mapping in our department from 2016 to 2019.

### 2.3 Data collection and procedures

For all patients, preoperative MRI (Achieva 3T, Philips Medical System, Netherlands BV) images were acquired, including DTI scans (TR/TE: 5,000/78 ms, voxel size of 2 × 2 × 2 mm^3^, 32 diffusion gradient directions, *b* value 1,000 s/mm^2^) and a 3D T1‐weighted gradient‐echo sequence with and without intravenous contrast agent (TR/TE: 9/4 ms, 1 mm^3^ iso‐voxel, Dotagraf 0.5 mmol/ml, produced by Jenapharm GmbH & Co. KG, Jena, Germany, phase‐encoding at rostral–caudal direction) in our neuroradiological department for quality control and stable scanning.

### 2.4 Language mapping and aphasia testing

The aphasia level testing was performed according to the Aachener aphasia test (AAT) for both groups (Biniek, Huber, Glindemann, Willmes, & Klumm, 1992). The handedness test was conducted according to Edinburgh Handedness Inventory (EHI; Oldfield, 1971).

nTMS language mapping was performed following the standard protocol used in clinical routine using a Nexstim eXimia NBS system (version 5.1.1; Nexstim Plc, Helsinki, Finland; Krieg et al., 2017; Sollmann, Fuss‐Ruppenthal, Zimmer, Meyer, & Krieg, 2018). Contrast‐enhanced T1 weighted images were used for neuronavigation. Stimulator output was set at 100% of the individual resting motor threshold. Stimulation was performed using 5 pulses at 5 Hz on individually predefined 46 targets according to the cortical parcellation system (Krieg et al., 2017; Sollmann et al., 2018). nTMS language mapping consisted of a baseline session without stimulation and a stimulating session with stimulation during the patient performing an object naming task (ONT), during which audios and videos were recorded for post‐hoc analysis. Each naming performance with stimulation was compared with the individual baseline to identify naming errors, which were categorized into five types: no‐response, performance, phonological paraphasias, semantic paraphasias, and neologism (Krieg et al., 2017; Sollmann et al., 2018). Stimulated sites with naming errors (positive nTMS stimulation regions [POS]) and without naming errors (negative nTMS stimulation regions [NEG]) were separated and exported as DICOM (digital imaging and communications in medicine) format files for further analysis. Mapping results were analyzed by both technicians and neurosurgeons. Durations of language mapping examinations were about 30 min per case.

### 2.5 Network construction

**FIGURE 1:**
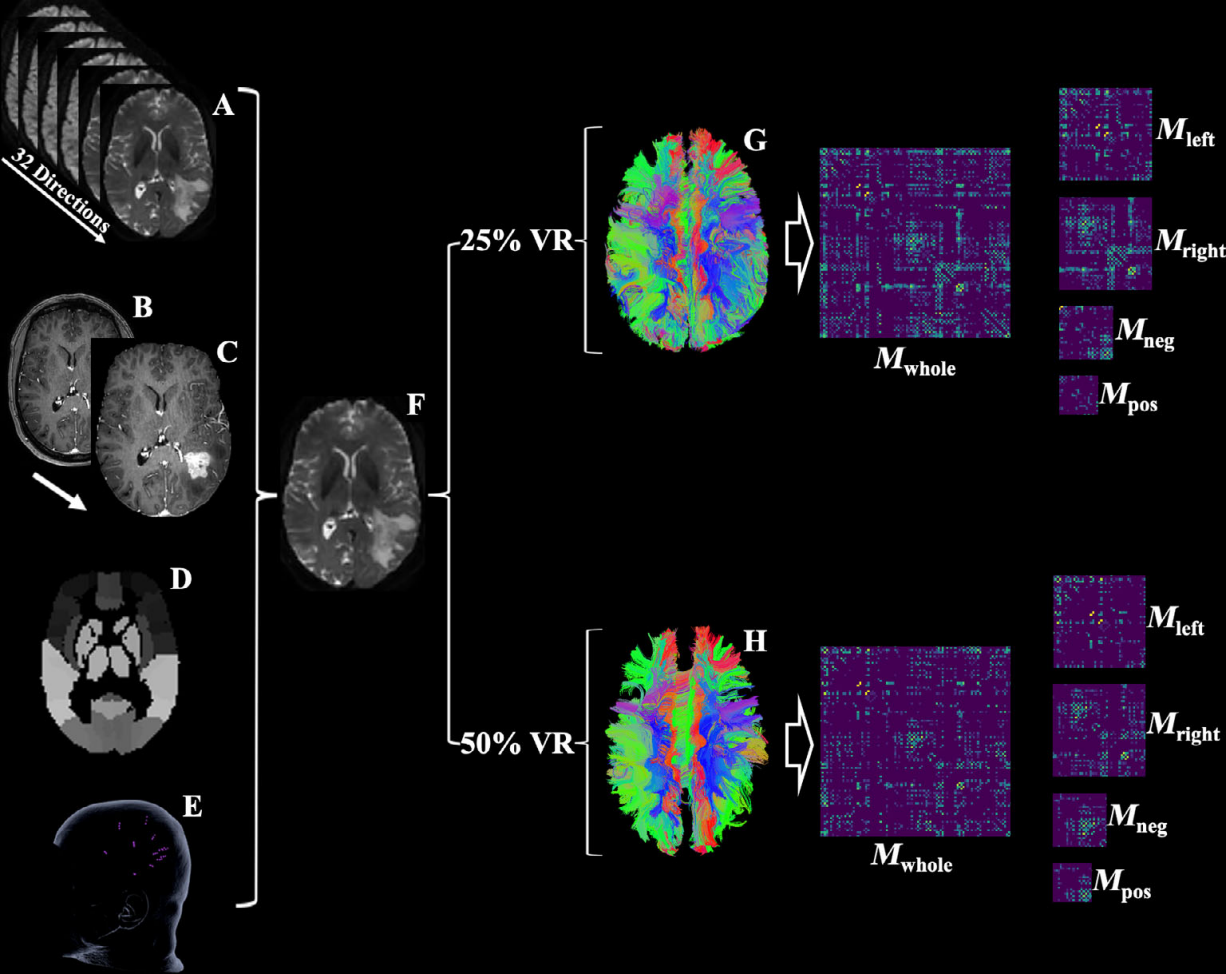
Workflow of the current study. This figure illustrates the process of network construction. DTI scans with 32 directions (A), T1 images with contrast (B), T1 images with contrast without skull and skin (C), the anatomic atlas template AAL90 (D), and nTMS language mapping images (E) were registered to a B0 image (F). A deterministic algorithm was used for fiber tracking after applying constrained spherical deconvolution (CSD). The minimal FA for each fiber to be visualized was identified, from which its visualization ratio (VR) was calculated. The fibers with VR values above the thresholds of 25% (G) and 50% VR (H) were respectively used to construct five matrices: *M*_whole_, matrix (*M*) derived from nodes from both hemispheres and edges (fibers) connecting them. *M*_left_ and *M*_right_, respective matrices with nodes from the left (*M*_left_) or the right hemisphere (*M*_right_) and intra‐hemispheric edges (fibers). *M*_pos_ and *M*_neg_, matrix with nodes from the positive language mapping regions and edges from their corresponding edges (fibers), and matrix with nodes from the negative language mapping regions and edges from their corresponding edges (fibers)

Contrast‐enhanced T1 images were skull‐striped using HD‐Bet (Isensee et al., 2019). In the next step, they were linearly co‐registered to b0 images derived from the DTI data set, and the POS and NEG stimulation sites derived from nTMS language mapping were transferred into the DTI space. The anatomic atlas template AAL90 (Tzourio‐Mazoyer et al., 2002) was co‐registered to the T1 image using the SyN algorithm from ANTs (https://github.com/ANTsX/ANTs; Avants, Epstein, Grossman, & Gee, 2008) and diffusion‐weighted b1000 images were linearly registered to the b0 image. The gradient vector table was rotated accordingly and corrected for eddy currents (Figure 1). Finally, nTMS POS and NEG sites, as well as AAL90 atlas locations, were co‐registered to the DTI space, and the total number of regions in the AAL90 anatomic template corresponding to POS or NEG points was counted for each subject.

Next, whole‐brain tractography was conducted through the Python library DIPY (Version 1.2.0, https://dipy.org; Garyfallidis et al., 2014), during which each voxel in the brain was used as region of interest (ROI; Figure 1). Then, constrained spherical deconvolution (CSD) was performed on the dataset (Tournier, Calamante, & Connelly, 2007). In the next step, a deterministic algorithm was applied to tractography with fractional anisotropy thresholds (FAT) starting from 0.0 continuously increased by 0.01 with a fiber length threshold (FLT) at 30 mm and stopping at a maximal FAT (FAT_max_). During this process, the maximal FA (fa_max_) for every single fiber was identified. The following formula calculated the visualized ratio (VR) for each fiber:

$$\mathrm{VR}=\frac{{\mathrm{fa}}_{\max}}{{\mathrm{FAT}}_{\max}}\times 100\%.$$

**FIGURE 2:**
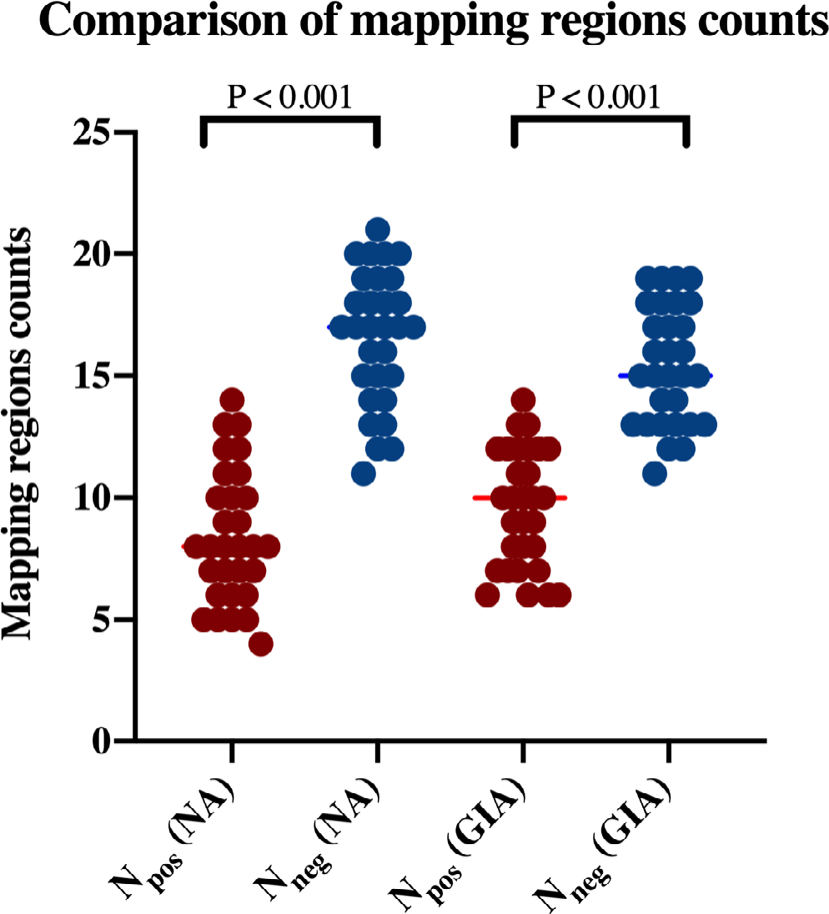
Comparison of mapping region counts. This figure presents counts of nTMS positive (*N*_pos_) and nTMS negative (*N*_neg_) mapping regions for patients with no aphasia (NA) and glioma‐induced aphasia (GIA). No significant intergroup differences were detected, while the intragroup analysis showed a lower count of *N*_pos_ compared to *N*_neg_ in both groups (*p* < .001)

**FIGURE 3:**
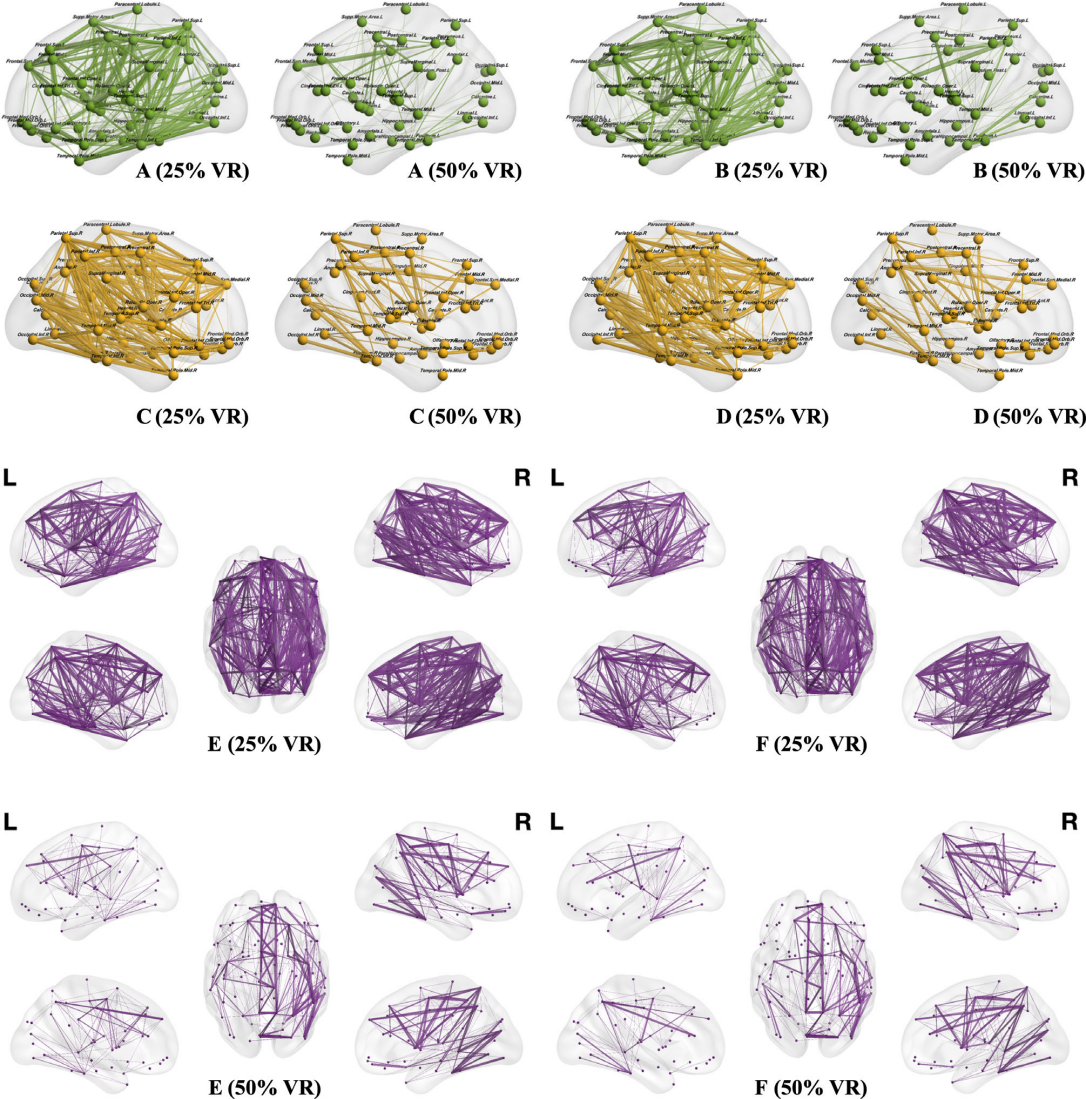
Nodes and connections from the whole brain, left hemisphere, and right hemisphere. This figure illustrates edges and nodes for matrices of the left hemisphere (*M*_left_—green), right hemisphere (*M*_right_—yellow), and both hemispheres (*M*_whole_—purple) under different visualization ratios (VRs). The connections tracked in <10 patients (33.3%) are not shown to improve the visualization. A larger thickness of the edges indicates higher intragroup prevalence of the respective edges. Connection density in the no aphasia (NA) group was observed to be higher than that in the glioma‐induced aphasia (GIA) group under both VRs

The tractography based on the fibers with VR above 25 and 50% were selected (Figure 1), from which the following five matrices (M) were constructed (Figures 2 and 3):

1. *M*_whole_: a matrix (M) at a size of 90 nodes × 90 nodes was created from both hemispheres based on the atlas AAL90 and their corresponding connections.
2. *M*_left_: a matrix based on nodes from the left hemisphere based on the atlas AAL90 and their corresponding connections at the size of 45 × 45.
3. *M*_right_: a matrix based on nodes from the right hemisphere based on the atlas AAL90 and their corresponding connections at the size of 45 × 45.
4. *M*_pos_: a matrix based on nodes from individual positive language mapping regions based on the atlas AAL90 and their corresponding connections.
5. *M*_neg_: a matrix based on nodes from individual negative language mapping regions based on the atlas AAL90 and their corresponding connections.

Since brain size is variable among individuals, connections between two regions in matrices containing more than three fibers were considered as connected and binarized to 1, otherwise as they were considered as disconnected and binarized to 0 (Figure S1).

The connectome properties, including AD, EG, and EL, were assessed in each of the five binarized matrices under VR thresholds of 25 and 50%, respectively, using algorithms from the NetworkX 2.5 library (https://networkx.org/) in Python 3.7 (https://www.python.org/; Latora & Marchiori, 2001). The differences of each property calculated for 25% VR and 50% VR thresholds were recorded for each group as AD‐diff, EG‐diff, and EL‐diff.

### 2.6 Connectome analysis and statistical analysis

The statistical analysis was performed using SPSS Statistic (IBM SPSS Statistics for Mac, Version 23.0. IBM Corporation, Armonk, NY) and GraphPad Prism (Version 8.4.3, San Diego, CA).

The chi‐square test was applied to compare the demographic data between both groups, including handedness, pathological diagnosis, tumor locations, and gender. Furthermore, independent *t*‐testing was applied to compare age and glioma size between the two groups.

For the analysis of mapping regions, the number of patients in each group with the same positively or negatively mapped regions was summarized, respectively. The intra‐group proportion of being positively or negatively mapped was calculated for each region in the mapping template. The nonparametric test was used to analyze the difference of the mapping points between the NA and GIA groups.

ANCOVA testing with covariates was used to compare network properties between the NA and GIA groups, consisting of AD, EG, EL, AD‐diff, EG‐diff, and EL‐diff. Correlation analysis was performed for different aphasia levels, properties, and the properties' alterations (AD‐diff, EG‐diff, and EL‐diff).

Figures were created in MATLAB (Version R2016b, Company; authorized license to TUM) using BrainNet Viewer (Version 1.7; https://www.nitrc.org/projects/bnv/; Xia, Wang, & He, 2013). A level of significance at *p* < .05 was set for all tests. FDR correction was applied for multiple comparisons.

## 3 RESULTS

### 3.1 Demographic analysis

Sixty subjects were enrolled from the database of patients receiving treatments in our department between 2016 and 2019, consisting of 30 patients in the NA group and 30 patients in the GIA group. Age was 57.7 ± 15.1 years for the NA group and 63.9 ± 12.4 years for the GIA group (*p* = .093). There were no significant differences between the two groups regarding handedness, gender, and World Health Organization (WHO) grading (Table 1). FAT_max_ was calculated for the NA (0.531 ± 0.065) and GIA (0.541 ± 0.063) groups. No significant differences were found between the two groups (*p* = .774).

**TABLE 1:** Comparisons on demographic data between NA and GIA group

Items	NA group	GIA Group	*p*
Gender			
Male	6	11	.152
Female	24	19	
Handiness			
Left	4	5	.718
Right	26	25	
Pathology diagnoses			
I–III	11	6	.152
IV	19	2	
Tumor sizes			
Average	2.4	4.7	.015^a^
*SD*	2.6	4.3	

*Note*: This table shows comparisons on patient characteristics between patients with NA (no aphasia) and GIA (glioma‐induced aphasia). Average values and standard deviations (SD) of tumor sizes are shown additionally.

a *p* < .05.

Notably, glioma size in the NA group (2.4 ± 2.6 cm^3^) was significantly smaller than in the GIA group (4.7 ± 4.3 cm^3^; Table 1; *p* = .015). However, glioma size did not correlate with aphasia levels in the GIA group (*p* = .060, *R* = .249). As the tumor size was different between the two groups, glioma size was regarded as a covariate for the analysis of covariance (ANCOVA) in the following comparisons between the two groups for investigating the performance of structural networks. In the GIA group, the tumor affected the precentral gyrus, frontal inferior gyrus, and Rolandic operculum more often (Table S1).

### 3.2 Analysis of nTMS mapping regions

**TABLE 2:** Intragroup proportion of each positive and negative language mapping region

NA group		GIA Group	
Proportion of each positive region	Proportion of each negative region	Proportion of each positive region	Proportion of each negative region
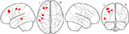	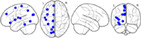	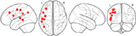	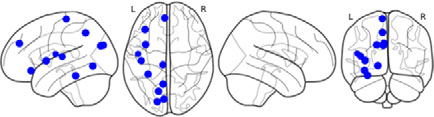
Middle frontal gyrus: 93.3% (28 cases) Precentral gyrus: 76.7% (23 cases) Middle temporal gyrus: 76.7% (23 cases) Postcentral gyrus: 70.0% (21 cases) Superior frontal gyrus: 66.7% (20 cases)	Paracentral lobule: 100% (30 cases) Fusiform gyrus: 100% (30 cases) Precuneus gyrus: 100% (30 cases) Lingual gyrus: 100 (30 cases) Cuneus gyrus: 100% (30 cases) Inferior frontal gyrus (orbital): 100% (30 cases) Insula: 100% (30 cases) Medial superior frontal gyrus: 96.7% (29 cases) Superior occipital gyrus: 96.7% (29 cases) Supplementary motor area: 86.7% (26 cases) Heschl's gyrus: 86.7% (26 cases) Rolandic operculum: 70.0% (21 cases)	Middle frontal gyrus: 90.0% (27 cases) Precentral gyrus: 83.3% (25 cases) Superior temporal gyrus: 80.0% (24 cases) Middle temporal gyrus:76.7% (23 cases) Postcentral: 70.0% (21 cases) Angular gyrus: 70.0% (21 cases) Supramarginal gyrus: 70.0% (21 cases) Inferior frontal gyrus (opercular): 70.0% (21 cases)	Insula: 100% (30 cases) Superior occipital: 100% (30 cases) Inferior frontal gyrus (orbital): 100% (30 cases) Precuneus gyrus: 100% (30 cases) Fusiform: 100% (30 cases) Cuneus gyrus: 100% (30 cases) Lingual gyrus: 100% (30 cases) Medial superior frontal gyrus: 96.7% (29 cases) Paracentral lobule: 90.0% (27 cases) Heschl's gyrus: 86.7% (26 cases) Rolandic operculum: 66.7% (20 cases)

*Note*: This table shows results of the intragroup analysis on overlapping positive and negative regions identified in more than 20 patients (66.7%) with NA (no aphasia) and GIA (glioma induced aphasia) after registration to the AAL90 template. All mapping regions located were in the left hemisphere. The chi‐square test was applied to identify mapped regions with significant differences between NA and GIA groups. The left supplementary motor area (SMA) was positively mapped in 4 cases of the NA group and 12 cases of the GIA group and with a significant difference between the NA and GIA group (*K* = 5.454, *p* = .019). The left angular gyrus is positively mapped in 13 cases of the NA group and 21 cases of the GIA group. The chi‐square test shows NA patients were significantly more often positively to be mapped in the left angular gyrus (*K* = 4.343, *p* = .037).

There were significantly less positive than negative stimulation sites in both the NA (Average counts of mapping regions: 16.600 (NEG) vs. 8.400 (POS); *p* < .001) and GIA (Average counts of mapping regions: 15.433 (NEG) vs. 9.567 (POS); *p* < .001) group (Table 2, Figure 2). More POS regions were found in the GIA group compared to the NA group (9.567 vs. 8.400, *p* = .083; Figure 2). In the GIA group, the total count of the POS regions (*R* = .226, *p* = .230) and NEG regions (*R* = −.226, *p* = .230) did not correlate with aphasia levels.

The *middle frontal gyrus* was most often mapped in both NA (28 patients; 93.3%) and GIA groups (27 patients; 90.0%) and without difference between the two groups (corrected chi‐square test, *p* > .05; Table 2). Besides, the *precentral gyrus*, *middle temporal gyrus*, and *postcentral gyrus* were positively mapped in more than 20 cases in both NA and GIA groups (Table 2). Positive stimuli in the *superior frontal gyrus* were detected in 20 NA patients (66.7%) and 16 GIA patients (53.3%). Only in the GIA group, *angular gyrus*, *supramarginal gyrus*, and *inferior frontal gyrus (Operculum)* were positively mapped in more than 20 patients. Regarding the comparison of positive mapping regions in the left hemisphere between the two groups, most of them were without statistical difference between the two groups. However, the *supplementary motor area* (SMA; NA: 4 patients; GIA:12 patients; *K* = 5.454, *p* = .019) and *angular gyrus* (NA:13 patients; GIA:21 patients; *K* = 4.343, *p* = .037) showed the most remarkable difference between GIA and NA patients through chi‐square testing (Table 2).

### 3.3 Intergroup network analysis

#### 3.3.1 Analysis of AD

**FIGURE 4:**
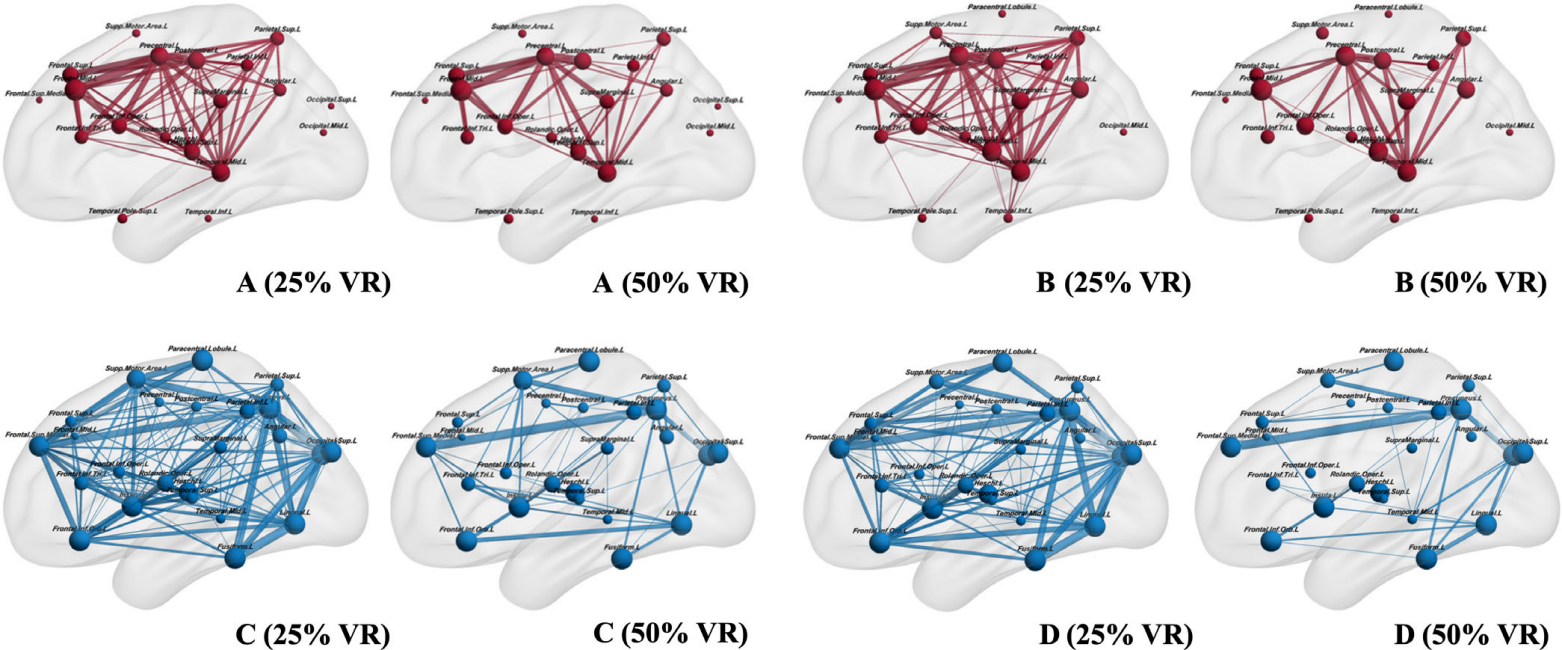
Nodes and connections based on nTMS mapping results. This figure illustrates nodes and edges for the matrices of nTMS positive mapping regions (*M*_pos_, red) as well as nTMS negative mapping regions (*M*_neg_, blue) in the left hemisphere under different VR setups. Fibers tracked in <3 patients (10%) are not shown as edges in the figures. A larger size of the nodes and edges indicates higher intragroup prevalence of the respective edges. There is a higher density of connections in patients without aphasia (NA) compared to patients with glioma‐induced aphasia (GIA) for 50% VRs. *M*_pos_ shows a higher density of edges at 25% VR in the GIA group [A (25% VR) vs. B (25% VR)]

Using thresholding at 25% VR and 50% VR, AD from different matrices (*M*_whole_, *M*_left_, *M*_right_, and *M*_neg_) in the NA group was higher than AD in the GIA group. *M*_pos_ was slightly higher in GIA patients at 25% VR (2.073 vs. 2.076; Figures 3 and 4, Table 3), while *M*_left_ was significantly higher in the NA group for 25% VR (5.430 vs. 4.827; *p* = .015) and 50% VR (1.833 vs. 1.434; *p* = .037). *M*_neg_ also showed a significant difference between groups under 25% VR (2.392 vs. 1.942; *p* = .042). The AD‐diff in the NA group was higher, except for the AD‐diff of *M*_pos_ (1.140 vs. 1.324; Figures 3 and 4, Table 3).

**TABLE 3:** Analysis on average degree, global, and local efficiency

Items	*M*_whole_	*M*_left_	*M*_right_	*M*_pos_	*M*_neg_
**25% VR**					
AD					
NA group	7.635	5.430	5.833	2.073	2.392
GIA group	7.215	4.827	5.509	2.076	1.942
*T*‐value	1.718	2.498	1.584	0.372	2.083
*p*‐value	.091	.015*	.119	.712	.042*
EG					
NA group	0.537	0.566	0.593	0.704	0.595
GIA group	0.520	0.533	0.578	0.705	0.532
*T*‐value	2.406	2.513	1.422	0.327	2.034
*p*‐value	.019*	.015*	.161	.745	.047*
EL					
NA group	0.717	0.716	0.732	0.682	0.633
GIA group	0.706	0.692	0.717	0.707	0.571
*T*‐value	1.517	2.093	1.456	0.315	1.027
*p*‐value	.135	.041*	.151	.754	.309
**50% VR**					
AD					
NA group	2.863	1.883	2.344	0.933	0.766
GIA group	2.457	1.434	2.126	0.753	0.523
*T‐value*	1.776	2.139	1.176	1.592	1.669
*p‐value*	.081	.037*	.117	.178	.101
EG					
NA group	0.330	0.297	0.371	0.437	0.227
GIA group	0.286	0.229	0.328	0.284	0.145
*T*‐value	1.701	1.392	2.344	2.187	1.506
*p*‐value	.094	.169	.023*	.033*	.138
EL					
NA group	0.367	0.332	0.373	0.338	0.174
GIA group	0.317	0.249	0.348	0.177	0.092
*T*‐value	1.591	2.340	0.662	2.586	1.513
*p*‐value	.117	.023*	.511	.012*	.136
**DIFF**					
AD‐diff					
NA group	4.772	3.547	3.489	1.140	1.626
GIA group	4.759	3.393	3.383	1.324	1.419
*T*‐value	0.494	1.509	0.978	0.400	1.112
*p*‐value	.624	.137	.332	.690	.271
EG‐diff					
NA group	0.207	0.269	0.222	0.267	0.368
GIA group	0.234	0.304	0.251	0.421	0.387
*T*‐value	1.452	1.348	1.437	2.891	0.425
*p*‐value	.152	.183	.156	.005**	.673
EL‐diff					
NA group	0.350	0.384	0.360	0.343	0.459
GIA group	0.389	0.442	0.369	0.530	0.479
*T*‐value	1.247	1.702	0.039	2.746	0.244
*p*‐value	.217	.094	.969	.008**	.808

*Note*: This table shows the average degree (AD), average global efficiency (EG), local efficiency (EL), and differences between a visualization rate of 25% (25% VR) and 50% (50% VR) for AD (AD‐diff), EG (EG‐diff), and EL (EL‐diff) of each matrix for patients with no aphasia (NA) and glioma‐induced aphasia (GIA). Most matrices in the no aphasia group (NA) show a higher AD than the glioma‐induced aphasia group (GIA), except for the matrices of nTMS positive regions (*M*_pos_) thresholding at a VR of 25%. The average EG and EL in the NA group were higher than that in the GIA group under 25% and 50% VR, except for EG and EL of *M*_pos_ under 25%VR, which were higher in GIA. The EG‐diff and EL‐diff were higher in GIA patients than in the NA patients for all matrices. Furthermore, results of the ANCOVA analysis on the average degree and efficiency of each matrix in both groups using tumor size as a covariate are shown. Under 25%VR, there were significant differences of EG in whole‐brain matrices (*M*_whole_), left hemispheric matrices (*M*_left_), and matrices of negative nTMS regions (*M*_neg_) between NA and GIA group, while EL in the M_left_ was significantly different between both groups. Under 50% VR, EG, and EL of *M*_pos_, and EL of *M*_left_ and EG of right hemispheric matrices (*M*_right)_ showed a significant difference between both groups. Regarding the changing levels (DIFF), EG‐diff, and EL‐diff of *M*_pos_ were significantly higher in the GIA groups.

* *p* < .05; ** *p* < .01.

#### 3.3.2 Analysis of the EL and EG of networks

At a threshold of 25% VR, EG, and EL in the GIA group were lower than in the NA group for the matrices *M*_whole_, *M*_left_, *M*_right_, and *M*_neg_. However, EG (0.704 vs. 0.705) and EL (0.682 vs. 0.707) of *M*_pos_ were higher in the GIA group (Table 3). For a threshold set at 50% VR, EG and EL in the GIA group were lower than in the NA group for all matrices (Table 3).

Regarding the comparison of *M*_left_, significant differences between the NA and GIA groups were found for EL in *M*_left_ at thresholds of 25% VR (0.716 vs. 0.692; *p* = .041) and 50% VR (0.332 vs. 0.249; *p* = .023), and for EG in *M*_left_ at a threshold of 25% VR (0.566 vs. 0.533; *p* = .015; Table 3). In the network of POS mapping regions, its EG (0.437 vs. 0.284; *p* = .033) and EL (0.338 vs. 0.177; *p* = .012) detected under 50% VR showed the most remarkable difference (Table 3).

There was a higher difference of EG (EG‐diff) and EL (EL‐diff) between both VR settings for the GIA group compared to the NA group (Table 3). Significant differences were found for *M*_pos_ regarding EG‐diff (0.267 vs. 0.421; *p* = .005) and EL‐diff (0.343 vs. 0.530; *p* = .008), which were higher in the GIA group (Table 3). However, comparisons of other matrices were without significance.

#### 3.3.3 Correlation analysis

The correlation analysis between aphasia levels and network properties included all cases from NA and GIA groups.

ADs from *M*_neg_ showed a negative correlation to the aphasia levels under both VR thresholds (25% VR: *R* = −.261, *p* = .044; 50% VR: *R* = −.281, *p* = .030). Furthermore, ADs of *M*_left_ correlated with aphasia levels under 50% VR (*R* = −.290, *p* = .025). No significances were detected on ADs from other matrices (Table 4).

**TABLE 4:** Correlation analysis of connectome properties and aphasia levels in all cases

Items	25% VR		50% VR		DIFF	
	*R*‐value	*p*‐value	*R*‐value	*p*‐value	*R*‐value	*p*‐value
AD						
*M*_whole_	−.156	.234	−.194	.137	−.011	.934
*M*_left_	−.232	.074	−.290	.025*	−.109	.414
*M*_right_	−.129	.327	−.099	.451	−.108	.418
*M*_pos_	−.004	.978	−.160	.221	.100	.454
*M*_neg_	−.261	.044*	−.281	.030*	−.074	.580
EG						
*M*_whole_	−.238	.067	−.240	.065	.259	.046*
*M*_left_	−.287	.026*	−.325	.011*	.281	.030*
*M*_right_	−.100	.448	−.345	.007**	.205	.116
*M*_pos_	−.152	.246	−.214	.101	.366	.004**
*M*_neg_	−.257	.047*	−.188	.151	.172	.189
EL						
*M*_whole_	−.012	.928	−.233	.073	.250	.054
*M*_left_	−.125	.347	−.296	.021*	.341	.008**
*M*_right_	−.059	.658	−.083	.529	.073	.580
*M*_pos_	−.108	.416	−.337	.008**	.365	.004**
*M*_neg_	−.112	.399	−.224	.085	.040	.760

*Note*: This table presents the results of the correlation analysis between aphasia levels in all cases and their connectome properties from different matrices consisting of the left hemisphere (*M*_left_), right hemisphere (*M*_right_), and both hemispheres (*M*_whole_), nTMS positive regions (*M*_pos_), and nTMS negative regions (*M*_neg_). *R* values and *p* values are shown. Under both 25% and 50% visualized rate (VR), average degrees (AD) from *M*_neg_ and global efficiency (EG) from *M*_left_ were correlated to aphasia levels. Thresholding at 25%VR, EGs from *M*_neg_ and *M*_left_ were correlated to aphasia levels. When thresholding at 50%VR, EG of *M*_right_, local efficiency (EL) of *M*_left_ and *M*_pos_ were correlated to aphasia levels. The differences of corresponding graphic properties between 25%VR and 50%VR (DIFF) regarding EG of *M*_whole_, *M*_left_, and *M*_pos_, and EL of *M*_left_ and *M*_pos_ were correlated to aphasia levels.

* *p* < .05; **p < .01.

Through the correlation analysis on efficiency, different graphic properties significantly correlated with aphasia levels at a global level, consisting of M_left_'s EG under 25% (*R* = −.287, *p* = .026) and 50% VR thresholds (*R* = −.325, *p* = .011) as well as the corresponding EG‐diff (*R* = .281, *p* = .030; Table 4). Furthermore, *M*_neg_'s EG under 25% VR (*R* = −.257, *p* = .047), *M*_right_'s EG under 50% VR (*R* = −.345, *p* = .007), and *M*_pos_'s EG‐diff (*R* = .366, *p* = .004) also significantly correlated with aphasia levels (Table 4). On the local level, ELs under 50% VR threshold from *M*_left_ (*R* = −.296, *p* = .021) and *M*_pos_ (*R* = −.337, *p* = .008) were negatively correlated with aphasia levels, while EL‐diffs from *M*_left_ (*R* = .341, *p* = .008) and *M*_pos_ (*R* = .365, *p* = .004) were positively correlated with aphasia levels (Table 4).

## 4 DISCUSSION

The present study focused on a graph‐based, joint structural–functional connectome analysis to investigate properties of language function‐related networks (*M*_pos_ and *M*_neg_) and anatomically related networks (*M*_whole_, *M*_left_, and *M*_right_) showing their differences in glioma patients with and without aphasia. SMA and the angular gyrus were positively mapped more frequently in GIA patients as compared to NA patients. Furthermore, the comparison of different network properties between the two groups proposes NEG network density to support language function and shows the importance of POS network efficiency in maintaining language function. It also suggests a new FT thresholding setup of VR.

### 4.1 The difference of nTMS language mapping regions between NA and GIA groups

Different language regions were identified by the means of nTMS in the NA and GIA group. Left SMA was more often positively mapped in GIA patients than in NA patients (*p* = .019). Previous studies found clinical manifestations of aphasia corresponding to the damage of the left SMA (Satoer, Kloet, Vincent, Dirven, & Visch‐Brink, 2014). SMA involves a superordinate controlling function during speech communication and language reception, particularly under elevated task demands, supporting the present results on language mapping (Hertrich, Dietrich, & Ackermann, 2016). The difference of overlapped ratios between two groups can be reasoned by the disconnection of SMA in the language network, which has been suggested in a study on functional connectivity properties of the language network in children with an autistic spectrum disorder, detecting a profound loss of functional connections between the SMA and modulatory control of the dorsolateral prefrontal region (Verly et al., 2014).

The angular gyrus is a critical area for language perception, as confirmed in previous studies (Hartung et al., 2020). In NA patients, there is a higher probability of the angular gyrus to be negatively mapped (30.0% in the GIA group and 56.7% in the NA group), supposing that there is a network of collaboration related to the angular gyrus (Papathanassiou et al., 2000). Hence, when nTMS targets the angular gyrus, the language‐related network in NA patients can better compensate for its inhibition.

### 4.2 Different network performance between NA and GIA group

The current study applied the deterministic algorithm to track fibers. Previous studies have shown that deterministic and probabilistic FT have respective advantages and disadvantages. Their sensitivity and specificity were affected by many factors, such as edema and/or hemorrhage around the tumor, resulting in uncertainty in the number of fibers obtained (Schlaier et al., 2017; Zhan et al., 2015). Therefore, this study was not only based on the exact number and volumes of connections but also on a binarized matrix to present the existence of connectivity among brain regions, which can meanwhile minimize the confounding effects of individual brain volume and tumor size in the analysis of network properties.

#### 4.2.1 Higher ADs in the NA group

Thresholding at both VRs, ADs from the matrices in the NA group were higher than in the GIA group, except for *M*_pos_ under 25% VR, which suggested that the NA group showed more connections of higher VR values (Table S2). ADs in *M*_left_ were smaller than in *M*_right_ even though they contained the same number of nodes. Gliomas located in the left hemisphere and its surrounding edema affected the intra‐hemispheric efficient cerebral connections. This corresponds to previous understandings of the reduction of intracerebral connections caused by glioma invasion. There were significantly more connections in the network based on negative mapping regions in the NA group, which also verified the importance of these areas in supporting language performance.

When combining the two groups of patients for correlation analysis, *M*_neg_'s ADs and aphasia levels showed a good correlation under both 25% and 50% VR thresholds (*p* = .044, *p* = .030, Table 4). More connections in NEG‐related networks were correlated to better language performances. It showed that the *M*_neg_'s density plays a major role in supporting language function, while we still do not know yet at which level of language production these regions act on. At the current point of view in neurosurgery, these negative regions were usually regarded as regions that can “safely” be resected without causing damages to language function and demonstrated a great consistency with intraoperative DCS (Ille et al., 2015a, 2015b; Picht et al., 2013). Previous studies using nTMS preferred to focus on positively mapped brain regions and their related subcortical structures due to language errors directly being induced by nTMS targeting these regions, which lead to an insufficient understanding of network structures and functions of NEG regions. Further studies are necessary to analyze those negatively mapped areas. However, the malignant growth of glioma also leads to difficulties in observing the long‐term effect after surgery in those negatively mapped regions.

The intergroup analysis showed no difference in the ADs from the POS‐based networks between NA and GIA groups. Notably, the count of POS regions was significantly smaller than the count of NEG regions, leading to a bigger size of *M*_neg_ compared to *M*_pos_. The ADs from *M*_pos_ were higher than the ADs of *M*_neg_ in the GIA group, which was not found in the NA group (Table 3). It demonstrates that the higher density in intra‐network connections within *M*_pos_ compared to *M*_neg_ in aphasia patients (Figure 4). It supports previous studies and indicates the enhanced connectivity profile within the POS‐related network (Sollmann, Zhang, Kelm, et al., 2020). Thus, POS regions might be the hubs of coordinating and processing information from different brain regions.

Previous studies have shown that gliomas led to a reduction in FA (Tropine et al., 2004). However, the impact of intracranial glioma on brain structural networks based on tractography constructed under different FA thresholds still has not been investigated. The current study found more fibers with a VR > 50% (Table S2) and smaller AD‐diff of *M*_pos_ in the NA group. Since the VR was mathematically based on the FA, it indicates that comprehensive fibers in *M*_pos_ present a downward trend of FA in GIA patients. Jiang et al. found that the functional defect resulting from lower limb amputees also displayed significant FA reduction in both the right inferior fronto‐occipital fasciculus and the commissural fibers connecting the bilateral premotor cortices (Jiang et al., 2015). Although experience in this field is scarce, our findings still demonstrate that glioma has a structural and functional influence on language function. Moreover, it also indicates the distinguished importance of the POS‐based network properties for language performance.

#### 4.2.2 Difference of efficiency between the two groups

Regarding the global performance, EG from *M*_whole_, *M*_left_, *M*_right_, *M*_pos_, and *M*_neg_ involved statistical differences under different VR thresholds. The correlation analysis on EGs also showed a significant correlation to aphasia levels (Table 4). The impairment of the cerebral structural organization by gliomas leads to reduced signal transmission and interaction efficiency at a global level. Na et al. (2018) analyzed white matter networks of adults suffering from brain tumors and found that their EG was significantly lower than in the healthy adult control group proving the long‐term effects of tumors on brain structures after resection. The structural network of patients suffering from mild cognitive impairment without anatomical structure damages also showed significantly lower EGs than normal subjects, and it was related to the functional score, which provided the correlation between changes in structural network properties and changes at a functional level (Berlot, Metzler‐Baddeley, Ikram, Jones, & O'Sullivan, 2016). Results of the current study regarding the EGs from brain structure‐based networks (*M*_whole_, *M*_left_, and *M*_right_) and nTMS‐related networks (*M*_pos_ and *M*_neg_) illustrate the importance of global network performance for language function.

Nevertheless, ELs from *M*_pos_ and *M*_left_ were the prominent properties regarding the difference in the performance of local regions between the NA and GIA group and negatively correlated to their aphasia levels (Table 3). Koenis et al. (2018) reported that higher local efficiencies of the tractography‐based network were related to higher intelligence and indicated efficiency of a local area to be related to the overall performance. Regarding neurosurgical treatment, this functional correlation indicates the necessity to avoid damaging positively mapped regions and their tracts, which has been demonstrated beneficial for resection planning and clinical outcomes (Raffa et al., 2018). In our study, ELs and EL‐diffs from left hemispheric and POS‐based networks correlate with aphasia levels (Table 4), further emphasizing the importance of preserving POS regions.

In the intragroup analysis in NA patients, EG‐diff (*p* < .001, corrected) and EL‐diff (*p* = .014, corrected) of *M*_neg_ were the largest among matrices (Figure S2). In the GIA intragroup analysis, however, EG‐diff (*p* < .001, corrected) and EL‐diff (*p* < .001, corrected) of *M*_pos_ were largest (Figure S2). Intergroup analysis showed that EG‐diff and EL‐diff of *M*_pos_ in the NA group were smaller than in the GIA group. This consistency of smaller *M*_pos_ efficiency changes at both local and global levels in the NA group compared to *M*_pos_ in the GIA group under different VR thresholds indicates its robustness for the preservation of language function. Gliomas mainly impacted *M*_neg_ in NA patients, while the tumor mainly impacted *M*_pos_ in GIA patients, indicating different POS and NEG functions. The global interference of gliomas in the distribution and performance of the brain's various networks leads to the clinical manifestation of different aphasia levels. Although the current study cannot explain the exact mechanism between global and local efficiency in language function, it proves that gliomas interfere with the information interaction of networks at local and global levels. Moreover, the alteration of connectome properties between different VR thresholds enhances functional performance investigations.

Alterations in the efficiency of various networks between NA and GIA patients reflect different capability information interactions and transmission in these brains. To some extent, this supports the theory of language function being based on the collaboration between dorsal and ventral streams as proposed in previous studies (Chang, Raygor, & Berger, 2015; Lou et al., 2019; Saur et al., 2008). It is essential to notice the importance of robustness of the POS‐related network performance in maintaining language function. The relationship between the dorsal and ventral streams and the degree of aphasia also reflects the impaired efficiency of information processing in cerebral networks impacted by tumors (Milner, 2017; van Polanen & Davare, 2015).

### 4.3 Limitations

The current study is based on the nTMS‐based language connectome applying a deterministic FT approach. However, some points should be taken into account when interpreting the results.

First, tumor sizes varied between the two groups. Yet, this is unavoidable concerning the induction of preoperative aphasia due to larger tumor volume. Moreover, glioma size did not significantly correlate to the GIA group's aphasia levels and was adopted in the ANCOVA analysis.

Second, data on cognitive impairment were not acquired in this study. Understanding the functional plasticity might further improve when combining the complexity of the language network with cognitive analysis. Yet, the application of combining cognition testing and nTMS mapping still needs to be further investigated.

Third, the ONT combined with nTMS language mapping cannot be performed by patients with complete aphasia. Thus, data of patients with extensive aphasia were not collected in the current study due to data consistency requirements. Furthermore, the ONT applied in nTMS mapping only involves the most basic language functions and is incapable of identifying logic and grammar‐related errors. POS and NEG regions found in ONT do not fully represent the complex organization of language function, which should be noticed when interpreting the results of the current study. Intracranial connectome changes in those patients could, however, further deepen the understanding of the language networks.

## 5 CONCLUSION

To the best of our knowledge, this is the first study to combine nTMS language mapping and connectome analysis to investigate the brain's glioma‐related functional changes. SMA is more likely to be involved in language production in GIA patients than in NA patients, indicating nTMS language mapping enables to investigate regions related to language superordinate controlling levels. Intracranial glioma interference causes differences in the distribution and performance of the brain's various networks mainly impacting *M*_neg_ in NA patients and *M*_pos_ in GIA patients. GIA patients were without sufficient *M*_neg_ compensation, this is related to their language dysfunction. nTMS mapping can identify unstable connectomes related to functional deficits such as aphasia.

Individualized FT threshold settings like VR in this study and changes in local and global connectome properties under different thresholds can reflect the lesions' impact on the language network. This new approach is suggested to identify language‐involved network components via a function‐specific connectome approach not only in glioma patients. Moreover, it could be applied to various diseases including stroke, traumatic brain injury, and neurodegeneration.

## CONFLICT OF INTEREST

The authors have no conflict of interest. Bernhard Meyer received honoraria, consulting fees, and research grants from Medtronic (Meerbusch, Germany), Icotec AG (Altstätten, Switzerland), and Relievant Medsystems Inc. (Sunnyvale, CA), honoraria, and research grants from Ulrich Medical (Ulm, Germany), honoraria and consulting fees from Spineart Deutschland GmbH (Frankfurt, Germany) and DePuy Synthes (West Chester, PA), and royalties from Spineart Deutschland GmbH (Frankfurt, Germany). Sandro M. Krieg is consultant for Ulrich medical (Ulm, Germany and Brainlab AG (Munich, Germany) and received honoraria from Nexstim Plc (Helsinki, Finland), Spineart Deutschland GmbH (Frankfurt, Germany), Medtronic (Meerbusch, Germany) and Carl Zeiss Meditec (Oberkochen, Germany). Bernhard Meyer received research grants and is a consultant for Brainlab AG (Munich, Germany). Sandro M. Krieg and Sebastian Ille are consultants for Brainlab AG (Munich, Germany). All authors declare that they have no conflict of interest regarding the materials used or the results presented in this study. All authors declare no other relationships or activities that could appear to have influenced the submitted work. This research did not receive any specific grant from funding agencies in the public, commercial, or not‐for‐profit sectors.

## AUTHOR CONTRIBUTIONS

Sebastian Ille, Benedict Wiestler, and Sandro M. Krieg conceived of the project. Haosu Zhang, Sebastian Ille, Benedict Wiestler, and Sandro M. Krieg designed the study. Sebastian Ille, Bernhard Meyer, Benedict Wiestler, and Sandro M. Krieg provided resources. Haosu Zhang, Sebastian Ille, Lisa Sogerer, and Axel Schröder collected the data. Haosu Zhang, Sebastian Ille, and Lisa Sogerer performed all analyses. Haosu Zhang, Sebastian Ille, Maximilian Schwendner, Sandro M. Krieg, and Benedict Wiestler wrote the manuscript. All authors discussed the results and contributed to the final manuscript. This study was officially registered prior to any patient enrolment in our local institutional registry, which can be accessed by the public as required by the ICMJE. Accordingly, the local ethics board reviewed and approved the trial plan (Ethics board of the Technical University of Munich).

## Supporting information


**Appendix S1**: Supporting Information

## Data Availability

Data and processes involved in this study are available upon reasonable request.
