# Supplementary material for: Elucidating the structural–functional connectome of language in glioma‐induced aphasia using nTMS and DTI
Source: Hum Brain Mapp. 2021 Dec 23;43(6):1836–49. doi: 10.1002/hbm.25757 (PMC8933329; doi:10.1002/hbm.25757)
Supplement: Supplementary file 1 — Appendix S1: Supporting Information [file HBM-43-1836-s001.docx]

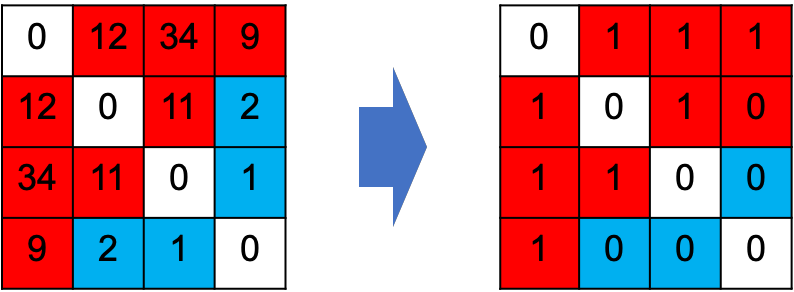


**Supplementary Figure 1.** Example for the binarization process. This connectivity matrix is calculated during fiber tracking and the number of fibers of the tract between every two regions is presented in the left subfigure. A tract with a count of fibers above the threshold of three fibers between any two regions will be considered as an effective connectivity between these two cerebral regions and therefore defined as 1. If a tract has a number of fibers below the threshold, it is regarded as an ineffective fiber connection between those two brain regions defined as 0. The binarized matrix, which is then used for further analysis is presented in the right subfigure.


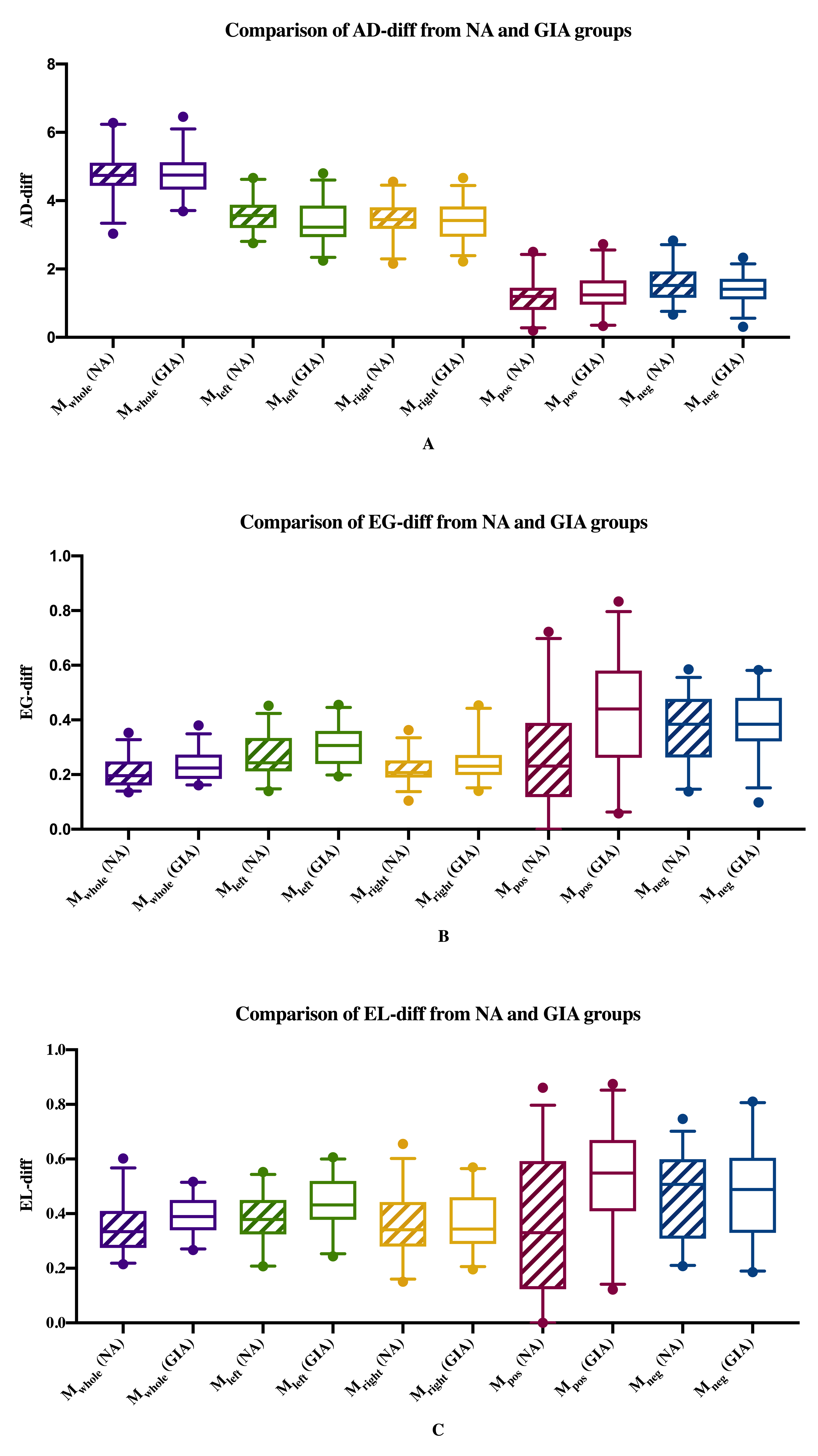


**Supplementary figure 2.** This figure presents intragroup comparisons on connectome properties, consisting of differences of average degrees (AD-diff) (A), differences of global efficiencies (EG-diff) (B), and differences of local efficiencies (EL-diff) (C). ANOVA analysis was tested by FDR correction. Subfigure A shows that AD-diffs from matrices of whole-brain network (M_whole_) were the biggest in both groups, the no aphasia (NA) group and the glioma-induced aphasia (GIA) group (P < 0.0001, corrected). In the GIA group, both EG-diff (P < 0.001, corrected) and EL-diff (P < 0.001, corrected) from matrices of positive nTMS points (M_pos_) showed the highest values. However, the highest EG-diff (P < 0.001, corrected) and EL-diff (P = 0.014, corrected) in the NA group were found in matrices based on networks of negative nTMS points (M_neg_) (subfigure B&C). M_left_ and M_right_ are respectively matrices based on left and right hemispheric regions.

**Supplementary Table 1. Chi-square test of tumor locations**

| **Region** | | **GIA** | **NA** | **K** | **P** |
| --- | --- | --- | --- | --- | --- |
| **Precentral gyrus** | invaded | 13 | 3 | *8.523* | *0.004 *** |
|  | not invaded | 17 | 27 |  |  |
| **Frontal inferior gyrus**  **(pars opercularis)** | invaded | 14 | 3 | *9.932* | *0.002 *** |
|  | not invaded | 16 | 27 |  |  |
| **Frontal inferior gyrus**  **(pars triangularis)** | invaded | 8 | 2 | *4.320* | *0.038 ** |
|  | not invaded | 22 | 28 |  |  |
| **Frontal inferior gyrus**  **(pars orbitalis)** | invaded | 8 | 1 | *6.405* | *0.011 ** |
|  | not invaded | 22 | 29 |  |  |
| **Rolandic operculum** | invaded | 15 | 6 | *5.934* | *0.015 ** |
|  | not invaded | 15 | 24 |  |  |
| **Supplementary motor area** | invaded | 7 | 1 | *5.192* | *0.023 ** |
|  | not invaded | 23 | 29 |  |  |
| **Olfactory** | invaded | 7 | 1 | *5.192* | *0.023 ** |
|  | not invaded | 23 | 29 |  |  |
| **Cingulum**  **(anterior)** | invaded | 9 | 1 | *7.680* | *0.006 ** |
|  | not invaded | 21 | 29 |  |  |
| **Parahippocampal gyrus** | invaded | 5 | 12 | *4.022* | *0.045 ** |
|  | not invaded | 25 | 18 |  |  |
| **Caudate** | invaded | 14 | 3 | *9.932* | *0.002 *** |
|  | not invaded | 16 | 27 |  |  |

**: < 0.05; **: < 0.01.*

The table presents the difference in tumor location between no-aphasia and glioma-induced aphasia patients.

**Supplementary Table 2. Intra-group ratio of the connections**

| **Items** | | **M_whole_** | **M_left_** | **M_right_** | **M_pos_** | **M_neg_** |
| --- | --- | --- | --- | --- | --- | --- |
| **NA** | **Average** | 0.369 | 0.332 | 0.393 | 0.472 | 0.293 |
|  | **Std** | 0.088 | 0.112 | 0.095 | 0.238 | 0.173 |
| **GIA** | **Average** | 0.335 | 0.291 | 0.378 | 0.361 | 0.258 |
|  | **Std** | 0.076 | 0.085 | 0.108 | 0.191 | 0.128 |
| **t-value** | | 1.590 | 1.607 | 0.587 | 1.997 | 0.894 |
| **p-value** | | 0.117 | 0.113 | 0.559 | 0.050 | 0.375 |

This figure presents the intra-group ratio regarding average values and standard errors (Std) calculated as the formula below:

$$\boldsymbol{Ratio=}\frac{\boldsymbol{Amount of}\boldsymbol{connections with visualized ratio (VR) higher than 50\%}}{\boldsymbol{Amount of}\boldsymbol{connections with visualized ratio (VR) higher than 25\%}}$$

It was higher in the no aphasia (NA) group than in the glioma-induced aphasia (GIA) group. Matrixes of the left (M_left_), right (M_right_), and both hemispheres (M_whole_) as well as for nTMS positive (M_pos_) and negative regions (M_neg_) are shown.
